# Relative Crystallinity of Plant Biomass: Studies on Assembly, Adaptation and Acclimation

**DOI:** 10.1371/journal.pone.0002897

**Published:** 2008-08-06

**Authors:** Darby Harris, Seth DeBolt

**Affiliations:** Department of Horticulture, University of Kentucky, Lexington, Kentucky, United States of America; University of Melbourne, Australia

## Abstract

Plant biomechanical design is central to cell shape, morphogenesis, reproductive performance and protection against environmental and mechanical stress. The cell wall forms the central load bearing support structure for plant design, yet a mechanistic understanding of its synthesis is incomplete. A key tool for studying the structure of cellulose polymorphs has been x-ray diffraction and fourier transform infrared spectroscopy (FTIR). Relative crystallinity index (RCI) is based on the x-ray diffraction characteristics of two signature peaks and we used this technique to probe plant assembly, adaptation and acclimation. Confocal microscopy was used to visualize the dynamics of cellulose synthase in transgenic Arabidopsis plants expressing a homozygous YFP::CESA6. Assembly: RCI values for stems and roots were indistinguishable but leaves had 23.4 and 21.6% lower RCI than stems and roots respectively. Adaptation: over 3-fold variability in RCI was apparent in leaves from 35 plant species spanning Ordovician to Cretaceous periods. Within this study, RCI correlated positively with leaf geometric constraints and with mass per unit area, suggestive of allometry. Acclimation: biomass crystallinity was found to decrease under conditions of thigmomorphogenesis in Arabidopsis. Further, in etiolated pea hypocotyls, RCI values also decreased compared to plants that were grown in light, consistent with alterations in FTIR cellulose fingerprint peaks and live cell imaging experiments revealing rapid orientation of the YFP::cellulose synthase-6 array in response to light. Herein, results and technical challenges associated with the structure of the cell wall that gives rise to sample crystallinity are presented and examined with respect to adaptation, acclimation and assembly in ecosystem-level processes.

## Introduction

Structural and morphological diversity is a striking feature of land plants. Lignin and a group of carbohydrate polymers (pectin, hemicelluloses and cellulose) form the scaffolding of the cell wall, which in turn are the building blocks for cell shape and morphogensis [1]. The crystalline nature of cellulose has been one of the central problems studied by polymer scientists. Cellulose microfibrils are the main structural component of plant cell walls and are formed of [1]–[4] linked b-D-glucosyl residues that are alternatively rotated by 180° along the polymer axis to form ribbon-like chains [2]. It has been established that each glucosyl residue has three hydroxyl groups, one of which is a hydroxymethyl group. The tight bonding capacity of the hydroxyl groups via hydrogen bonding are critical to determining how the crystal structure of cellulose forms and also in directing important physical properties of cellulose materials [3]. The chains of glucosyl residues in the fibril periodically fail to coalesce into an ordered crystalline structure; these amorphous zones along the fibril length are recognized as possibly serving the association between hemicellulose and cellulose fibrils [4]. In elongating plant tissue, cellulose deposition is generally considered to occur perpendicular to the axis of elongation, constraining lateral swelling (due to internal turgor pressure) and allowing longitudinal or anisotropic cell expansion [5]. A surprising gap in our understanding concerns fibril length and what controls it, with current estimates ranging from 300 to 15,000 glucan units [6]. Another unresolved question regarding cellulose biosynthesis is whether cellulose fibril orientation, length or crystallinity may provide differential biomechanical properties to certain cell and tissue types and how this may correspond to a specific ecological niche?

Study of the overall variation in plant function have largely focused on traits such as foliar stoichiometry [7], specific leaf area [8], seed and seedling characteristics [9], leaf area/dry mass (specific leaf area)[10], wood specific gravity [11], stem diameter [8] and the relationship between stem and branch wood specific gravity [12]. This building number of key functional traits is aimed at providing botanists with the ability to characterize the differential body plan and biomass allocation of plants with respect to their ecological niche [13]. Do plants adapt to environmental stimuli by regulating the density, orientation and/or biomechanical properties of cellulose fibrils in differentiated tissues? Recent studies of cellulose biosynthesis have shown that the upper hypocotyls of dark grown Arabidopsis seedlings expressing a functional YFP tagged cellulose synthase6 (CESA) have a transverse oriented CESA array. Yet, when this array is exposed to light the array rapidly changes (within 20 min) to a longitudinal array [14] suggesting that elongating plants that are searching for light are very rapidly able to alter the biomechanics of their cellulose array. The orientation of CESA motility [14], [15] appears to be guided by underlying cortical microtubules. Moreover, the site of CESA insertion at the plasma membrane occurs in a non-random pattern suggestive of regulation [16]. It has recently been found that the plant may have a cell wall sensing mechanism, *THESEUS1,* to provide transcriptional feedback on the integrity of the cell wall [17]. Recent discoveries at the cellular level suggest much is to be learnt about the regulation and plasticity of cellulose synthesis and its contribution to morphogenesis. The necessary body plan and biomass allocation properties of plants that proliferate under certain selection pressures such as growing at a rainforest floor have been found to differ dramatically from those that adapted to the upper canopy environment [18]. Land plant ecology on the basis of functional traits would thus suggest that plants adapt their biomechanical structure [19], [20]; this hypothesis was to be addressed in the current study with respect to the structure of cellulose.

Experiments described herein were designed to determine whether relative crystallinity of plant biomass samples was capable of responding to environmental cues. A central tool for polymer scientists studying the structure of cellulose polymorphs has become x-ray diffraction [21]–[22], which has been shown to reflect the degree of polymerization [23] as well as structure [24]–[29]. Adopting published methods for the calculation of a relative crystallinity index (RCI, 22) as well as results gathered using synthetic cellulose (Simacell, 22 or Avicel, 27), RCI was determined using x-ray diffraction (XRD) in various plant samples. RCI determination can be influenced by the preferred orientation of cellulose crystallites in a sample [27] or the size and surface area of the cellulose crystallites [30]. Hence it is important to note that the RCI parameter is relative to the portion of crystalline cellulose in the sample, thus defined as crystallinity of the sample rather than absolute crystallinity of the cellulose itself. Problems in crystallinity determination for wood samples from Norway spruce and Scots pine by transmission x-ray diffraction suggest that cellulose properties of size, orientation and crystalline:amorphous ratios are important considerations when assessing diffractogram differences [27]. Plant response to abiotic physical stresses such as wind was assessed by RCI and in more detail etiolation was assessed by x-ray diffraction, YFP::CESA6 experiments and FTIR analysis. RCI was measured in a study of 35 diverse plant species, spanning liverworts to several C4 grasses. The RCI values of root, shoot and leaf were compared to assess differences among plant assembly components. Finally, these results are discussed with respect to technical challenges and ecological and biomechanical context.

## Results

### Determining crystallinity index for plant biomass

**Figure 1:**
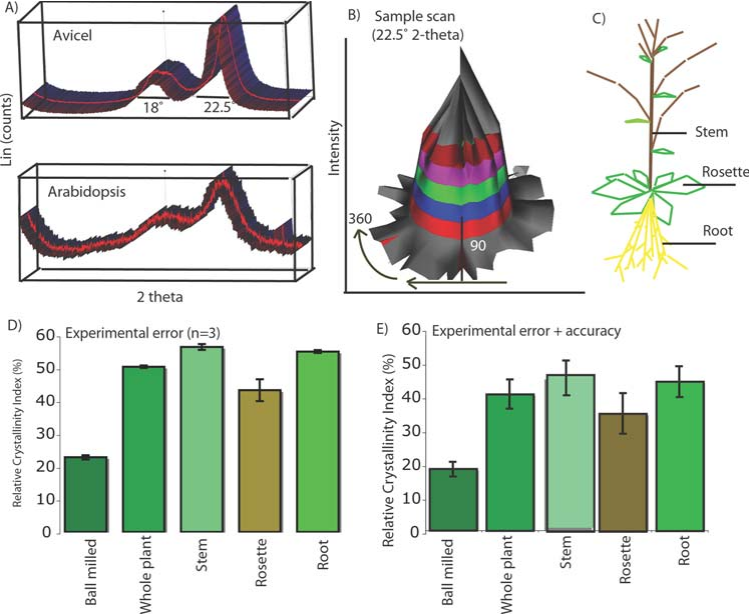
X-ray diffraction and plant ontogeny. A) Synthetic crystalline cellulose (Avicel) displayed a diffractogram pattern consistent with diffractogram from plant biomass derived from Arabidopsis whole plant biomass (B) Schematic of the sample scan used to estimate the experimental accuracy of pressed biomass at 22.5° 2-theta. Experimental accuracy was determined to be 8.99% (C) Roots, leaves and shoots from Arabidopsis plants were sub-sampled and analyzed by XRD showing variability of RCI between tissues. (D) RCI values for ball milled samples, whole plant sample and stem, rosette (leaf) and root values. *Rosette RCI was significantly lower than stems or roots (P<0.001 ANOVA) whereas stems and roots were not significantly different. (E) RCI values for ball milled samples, whole plant sample and stem, rosette (leaf) and root values with experimental accuracy included.

Previous methods for estimating the crystallinity of cellulose [21]–[29] were adapted to plant biomass samples (Figure 1A and 1B, Methods and Materials). Initially, synthetic cellulose (Avicel) was pressed into a custom-built boric acid base using 40,000 pounds per square inch (psi) and analyzed using Bragg-Brentano reflective geometries. X-ray diffraction pattern (Figure 1A) showed consistent signature peak distribution with previous published reports [21]–[29] and an average relative crystallinity index for synthetic crystalline cellulose of 65.8±8.12%. The experimental accuracy was approximated by determining the noise in the diffractogram using a *Phi* and *Chi* scan (360° rotational by 90° in an arc) of the sample, creating a intensity/spatial orientation plot at 22.5° 2-theta (Figure 1B). However, this value does not take into account the possibility that texture of the sample influences RCI. It was found to be extremely challenging to use the leaf and non-woody biomass samples for the analysis of transmission geometries. Most technically challenging was mounting the sample, x-ray penetration through the sample and orientation of the fiber axis (some of which is random in leaf cells). Our attempts centered on the use of pressed potassium bromide (KBr) into a 7 mm diameter mold to create an opaque disk with the sample embedded, but maintaining any control over fiber orientation was not achieved. Also, the x-rays were not penetrating the disk well enough to provide enough intensity of the symmetrical transmission geometries (even at 110 and 200 A our peaks were very low intensity with rocking in the 110 A range required to create peak resolution). A lack of texture analysis in the samples by transmission geometries does not allow us to predict the preferred orientation of crystallites within the samples, nor the size or density of crystallites. Rather, RCI provides relative value for the reflective geometry of sample crystallinity.

Experimental samples were packed and analyzed in triplicate and the error estimated between sample replicates was added to the estimated accuracy error. Arabidopsis whole plant samples were measured in 12 independent samples and the value remained between 47.5% and 49.8%, suggesting that the relative estimate was reproducible, but only provided a relative crystallinity value of the sample not absolute crystallinity. Nonetheless, total error may still be underestimated, considering the results of [27] who found that texture considerably affected determination of the experimental accuracy.

### RCI measurement of root, stem and rosette tissue in Arabidopsis

Whole plant *Arabidopsis thaliana* Columbia tissue from a combined 50 plant crop grown at 22°C under 16:8 light: dark cycle demonstrated that the average crystallinity of cellulosic biomass was 48.49% with a total RCI error of 4.71, which gave a total error of 9.47%. These data were consistent in several independently grown crops and gathered from 12 experimental replicates. Treatment of sample with three hot ethanol (70%) washes to remove chlorophyll and other cellular debris did not alter the RCI measurement nor did the drying samples at 37°C for 7 days compared with drying at 80°C (data not presented). *Arabidopsis thaliana* Columbia plants grown to maturity (at which time siliques had filled out containing mature seed) were harvested and divided into roots, stems and leaves (Figure 1C). Average RCI value for 3 independent measurements for stem was 54.79%±0.83, leaf (rosette RCI = 41.99%±3.21) and root (RCI = 53.42%±0.42) (Figure 1D). However, taking into consideration the experimental accuracy the total error was considerably greater with stems (54.79%±5.76), leaf (rosette RCI = 41.99%±6.98) and root (RCI = 53.42%±5.22) (Figure 1E). Relative crystallinity index of the Arabidopsis leaf tissue was 23.6% and 21.4% lower that shoot and root tissue respectively (Table 1). The volume fraction of cellulose in the leaf tissue (22% of total biomass) was approximately 36% lower than both stems (35%) and roots (32%).

**Table 1:** Plant species analyzed within this study.

Common name	Botanical name	Emergence	Tissue	RCI (avg)	St. Dev	Exp rep No.	Exp Accuracy	Total Error	Total error %
Avicel			Synthetic cellulose	65.80	2.20	2	1.97	4.17	6.3
Liverwort	*Marchantia polymorpha*	Ordovician	Leaf	18.18	0.89	2	0.91	1.80	9.9
Lance leaf sundew	*Drosera adelae*	Cretaceous	Leaf	31.75	1.04	2	1.59	2.63	8.3
Monkey puzzle	*Araucaria araucana*	Jurassic	Leaf	33.33		1	1.67	1.67	5.0
Buddhist pine	*Podocarpus macrophyllus*	Permian	Leaf	33.93		1	1.70	1.70	5.0
Australian tree fern	*Sphaeopterus cooperi*	Mid devonian	Leaf	35.24	0.76	2	1.76	2.52	7.2
Horsetail	*Equisetum hyemale*	Carboniforous	Leaf	37.50	0.45	2	1.88	2.33	6.2
Burro tail	*Sedum morganianum*	Carboniforous	Leaf	40.00	0.49	2	2.00	2.49	6.2
Cycad Queen Sago	*Cycas circinalis*	Jurassic	Leaf	41.11	0.89	2	2.06	2.95	7.2
Cyrus spp	*Epiphyllum oxypetalum*	Cretaceous	Leaf	41.76		1	2.09	2.09	5.0
Plumosa fern	*Asparagus setaceus*	Cretaceous	Leaf	46.15		1	2.31	2.31	5.0
Arabidopsis	*Arabidopsis thaliana*	Devonian/carboniforous	Whole plant	48.94	0.31	12	2.45	2.76	5.6
Cast iron plant	*Aspidistra elatior*	Cretaceous	Leaf	48.75		1	2.44	2.44	5.0
Switch grass Kan Low	*Panicum virgatum*	Cretaceous	Leaf	55.54	0.64	3	2.78	3.42	6.2
Switch grass Cave Rock	*Panicum virgatum*	Cretaceous	Leaf	55.54	0.40	3	2.78	3.18	5.7
Sweet Sorghum	*Sorghum bicolor*	Cretaceous	Leaf	55.54		1	2.78	2.78	5.0
Arundo donax	*Arundo donax*	Cretaceous	Leaf	53.57		1	2.68	2.68	5.0
Giant Miscanthus	*Miscanthus giganteus*	Cretaceous	Leaf	57.9		1	2.90	2.90	5.0
Sweet Miscanthus	*Miscanthus saccharifolia*	Cretaceous	Leaf	52.2		1	2.61	2.61	5.0
Flame Miscanthus	*Miscanthus sinescens*	Cretaceous	Leaf	55.2		1	2.76	2.76	5.0
Eastern gamagrass PMK24	*Tripsacum dactyloides*	Cretaceous	Leaf	55.7		1	2.79	2.79	5.0
Eastern gamagrass Meade	*Tripsacum dactyloides*	Cretaceous	Leaf	55.9		1	2.80	2.80	5.0
Eastern gamagrass Jackson	*Tripsacum dactyloides*	Cretaceous	Leaf	56.3		1	2.82	2.82	5.0
Indian gamagrass Cheynne	*Sorghastrum nutans*	Cretaceous	Leaf	52.1		1	2.61	2.61	5.0
Big Bluestem KAW	*Andropogon gerardii*	Cretaceous	Leaf	58.5		1	2.93	2.93	5.0
Big Bluestem KYAG09	*Andropogon gerardii*	Cretaceous	Leaf	55.6		1	2.78	2.78	5.0
Arabidopsis	*Arabidopsis thaliana*	Cretaceous	Ball Milled	22.14	0.22	2	1.11	1.33	6.0
Arabidopsis	*Arabidopsis thaliana*	Cretaceous	Whole plant (Grind)	48.94	0.36	3	2.45	2.81	5.7
Arabidopsis	*Arabidopsis thaliana*	Cretaceous	Stem (Mature)	54.79	0.83	3	2.74	3.57	6.5
Arabidopsis	*Arabidopsis thaliana*	Cretaceous	Rosette (Mature)	41.99	3.21	3	2.10	5.31	12.6
Arabidopsis	*Arabidopsis thaliana*	Cretaceous	Root (Mature)	53.42	0.42	3	2.67	3.09	5.8
Arabidopsis	*Arabidopsis thaliana*	Cretaceous	Stem+wind (3 wk)	52.08	1.23	3	2.60	3.83	7.4
Arabidopsis	*Arabidopsis thaliana*	Cretaceous	Stem−wind (3 wk)	55.80	0.38	3	2.79	3.17	5.7
Arabidopsis	*Arabidopsis thaliana*	Cretaceous	Leaf+wind (3 wk)	42.02	0.88	3	2.10	2.98	7.1
Arabidopsis	*Arabidopsis thaliana*	Cretaceous	Leaf−wind (3 wk)	42.64	0.98	3	2.13	3.11	7.3
Pea	*Pisum sativum*	Cretaceous	Hypocotyl Dark (7 d)	23.07	1.54	3	1.15	2.69	11.7
Pea	*Pisum sativum*	Cretaceous	Hypocotyl Light (7 d)	26.86	1.18	3	1.34	2.52	9.4

Common and botanical names, evolutionary emergence, tissue used in analysis, relative crystallinity index (RCI), number of samples replicates, standard error among replicates where applicable, experimental accuracy and total error.

### Crystallinity index among a spectrum of land plants

**Figure 2:**
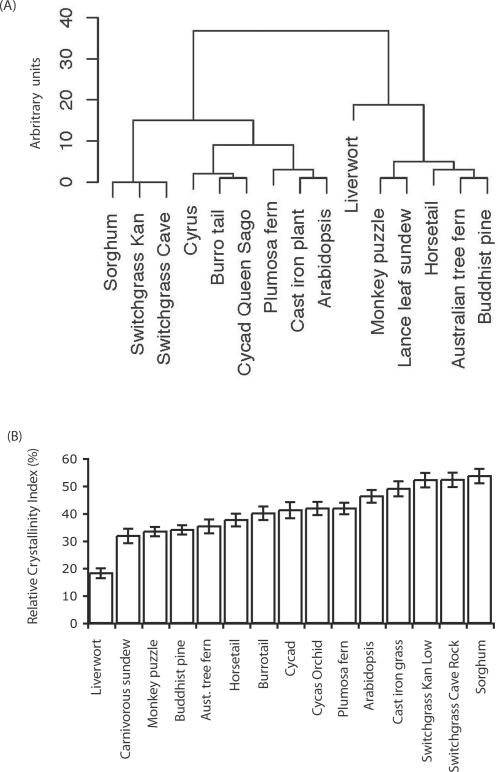
RCI values and their hierarchical cluster dendogram for a foliar samples from diverse range of species indicates a large degree of variability. A) Samples were clustered based on values for their RCI (B).

Foliar samples of 35 different plants were collected, oven dried and the RCI measured by XRD (Table 1, Figure 2A and 2B). It was evident that crystallinity measurements varied greatly among species assayed. The grasses had the highest RCI (58.5%) of all species tested and liverwort (*Marchantia polymorpha*), had the lowest RCI (18.8%). Of a collection of 22 grass species, the range in RCI was determined to be 51.1% to 58.5%, which was higher than leaves from all other species and more similar to values acquired from the stem of Arabidopsis. Other noteworthy observations were the similarity of RCI values for *Podocarpus macrophyllus* and *Araucaria araucana,* which are both of the pinophyta phylum. *Cycas circinalis* (Cycad), *Sedum morganianum* (Burro tail), *Equisetum hyemale* (horsetail) and *Epiphyllum oxypetalum* (Orchid) also displayed a similar RCI value ranging from 37.5 to 41.7%. The carnivorous plant *Drosera adelae* (lance leaf sundew) measured the second lowest RCI of 31.8%. Indeed, other plants with a similar morphology to the lance leaf sundew but very different metabolism such as Arabidopsis displayed higher RCI values (Table 1, Figure 2).

### Relative crystallinity, analysis of cellulose reorientation using YFP::CESA6 in dark to light conditions and FTIR spectral analysis of etiolated versus light-grown pea hypocotyls

**Figure 3:**
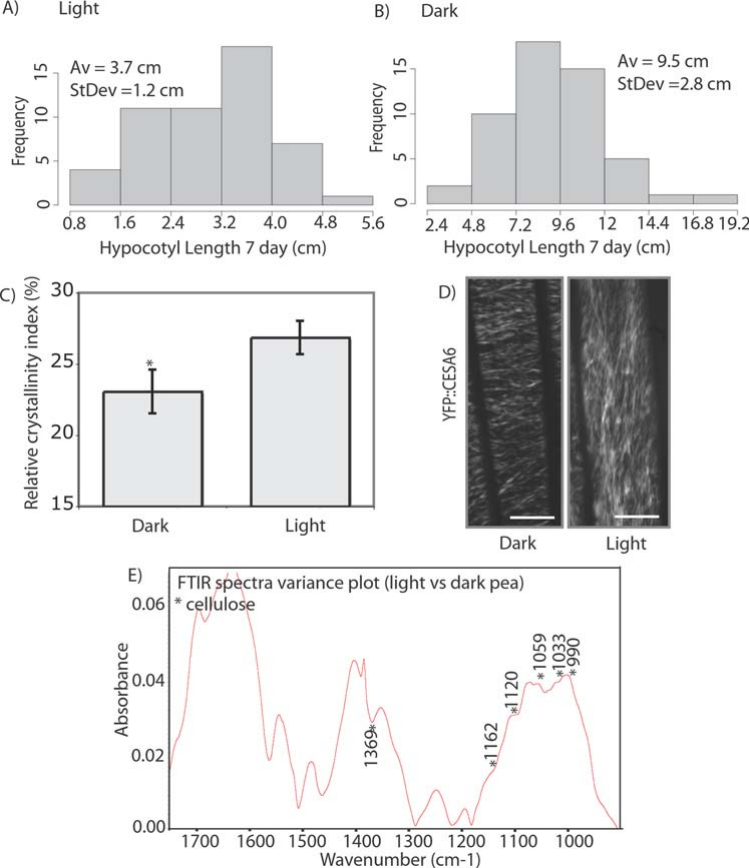
Hypocotyls lengths and RCI of etiolated pea (*Pistum sativum*) seedlings compared with light grown seedlings were significantly different. (A) Histogram of hypocotyl length for light grown and (B) dark grown. Dark grown hypocotyls had a significantly lower RCI that light grown (C) (*P<0.001 ANOVA). (D) Arabidopsis plants expressing YFP::CESA6 displayed a transverse orientation under dark conditions and longitudinal array under light conditions, images are time averages of 61 frames taken 10 sec apart for 10 min. Maximal linear trajectories particles was 14.66 mm for dark and 26.8 mm for light grown. Scale bar = 10 mm (E) FTIR spectral variance plot of light versus dark grown pea plant samples.

Dark-grown versus light grown seedlings invest a greater proportion of cellular energy into seeking light (elongation) and maximizing, capturing and transmitting light [31]. Shade conditions have also been shown to increase Young's modulus in petioles resulting in greater tensile strength [32]. Because the plant modified its body plan under dark growing conditions, this provided an opportunity to test whether RCI changed. Seven day old, dark-grown etiolated pea (*Pisum sativum*) seedlings displayed no pigmentation of hypocotyls and the tissue geometry and morphology were perpendicular to the growth media horizontal surface. Seedling heights were variable as demonstrated by the average height frequency graph (Figure 3A and 3B). The average height of dark-grown seedlings (9.5±2.8 cm) was significantly greater than light-grown seedlings (3.7±1.2 cm P<0.001, Wilcoxan Ranked Signed Test). Leaf and root tissue was separated and discarded and light and dark-grown hypocotyls were then oven dried, pressed and packed into boric acid for analysis by XRD. The corresponding RCI measurement of dark grown hypocotyls was 23.07±1.54% (n = 3) with an estimated experimental accuracy of 2.07 (total was 3.61 or 15.64%). Based on sample replicates, this value was significantly lower (P<0.05 ANOVA) than measurements made in light grown hypocotyls 26.86±1.17%, however considering the level of experimental accuracy measured using a scan of the sample (Figure 1B), the determination of significance was not substantiated (Figure 1C and D, Figure 3C, Table 1).

Because it is postulated that each visible YFP::CESA6 puncta represents an intact cellulose synthase rosette, we performed measurements of the YFP::CESA6 dynamics before and after light exposure. Dark grown seedlings were exposed to a light source for 5 min and then reimaged 30 minutes later (Figure 3D). Firstly, we confirmed orientation shift from within the range of 180°±20 to 90°±20 [14]. Secondly, measurement of particle velocity of membrane bound YFP::CESA6 particles between transverse (dark) and longitudinal (light) oriented arrays were not significantly different (274 nm.min^−1^±34 and 268 nm.min^−1^±41, Wilcoxan Ranked Signed Test). Thirdly, average track lengths in the transverse array were 11.33±1.46 µm compared with 14.1±2.9 µm (P>0.05 ANOVA) for the longitudinal array. Maximum track lengths were 14.66 µm in the transverse array compared with 26.8 µm after exposure to the light in the longitudinal array. The combined length of a cellulose track in a given time consists of multiple particles in a 61 frame time lapse image. Fourth, the number of individual CESA puncta in a given track were compared by the calculation: CesA_D_ = T_L_/P_n_ where CESA_D_ is CESA density along a track, T_L_ is the track length over 61 frames (µm) and P_n_ is the average particle number along that track. An average of 12.02±2.92 particles per strand (n = 47 strands) were counted in transverse arrays compared with 13.36±4.30 particles per strand (n = 47 strands) in longitudinal arrays (P>0.05, Wilcoxan Ranked Signed Test). Average CESA density (particles. µm^−1^) along a single track was lower in the transverse array (0.86±0.08 particles.µm^−1^) than the longitudinal array (1.05±0.13 particles. µm^−1^) but again not significantly lower (P>0.05, ANOVA). Hence, orientation was the only significant change in the cellulose array.

The FT-IR spectra from the cell walls showed a clear separation of the light grown pea hypocotyls compared with dark grown seedlings using an analysis of variance spectra generated from four replicates. Spectral variance in the FTIR data (Figure 3E) within the polysaccharide fingerprint region was difficult to assign to a single cell wall polymer and was more likely a reflection of cell wall reorganization. In the variance spectrum, there were distinct peaks in the FTIR spectrum that have been defined as arising from cellulose [33](990, 1033, 1059, 1120, 1152 and 1369 cm^−1^) (Figure 3E). In addition, amide-I absorption peak at 1673 cm^−1^ (Figure 3E) may reflect higher protein content.

### Relative crystallinity under artificial high wind conditions in the stems and leaves of Arabidopsis thaliana

Plant responses to the effect of high wind have been well reported (reviewed in 19). In particular wind treatment is known to decrease Youngs modulus [19]. Cellulose is the main structural component of the cell wall, yet effects of high wind on the crystalline physical properties of plant samples by XRD have not been established. A constant flow of wind energy was passed over 7 day old Arabidopsis plants in a crude wind tunnel within a growth chamber. Plants exposed to constant high wind conditions were dwarfed and leaf area decreased consistent with previous reports [20]. The RCI values for stems tissue sampled from plants exposed to high wind (4.3 m.sec^−1^)(RCI = 52.08±1.23%) were significantly lower (among experimental replicates (P<0.001, ANOVA) than stems from plants not exposed to wind (RCI = 55.8±0.38%) (Table 1). Yet again, given the estimated experimental accuracy of 11.2 and 9.52% respectively, a conclusion regarding the significance of these results cannot be substantiated. Particularly since in leaves the RCI measure under wind exposure was not significantly lower than from those no wind experiments based just on sample replicates alone (P>0.05 ANOVA).

### Leaf Mass Per Unit Area and Leaf Length Measurements Correlated With RCI

**Figure 4:**
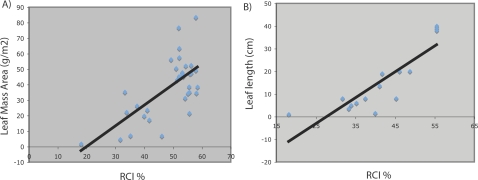
Pairwise comparison of foliar traits with RCI (A) RCI versus leaf mass per unit area B) RCI versus leaf length.

Species analyzed in this by RCI study were correlated against measurements of leaf mass per unit area (LMA) and leaf length. The correlation between RCI and LMA demonstrated the as LMA increased, so did the value for the RCI of the biomass sample (Figure 4A). Individual pairwise analysis of measurements of leaf length with RCI also showed positive correlation between these traits (Figure 4B).

## Discussion

All plant cells are surrounded by a rigid cell wall that constrains internal turgor pressure and yet must yield in a controlled and organized manner to allow the cell to grow and acquire a specific shape [1]. The major load-bearing constituent of the higher-plant cell wall is cellulose, which forms crystalline fibrils that are often highly organized with respect to cell shape and growth pattern [1]. Yet, the microfibril chains are thought to periodically fail to coalesce into an ordered crystalline structure and thus may have variation in the frequency of amorphous regions within the crystalline fibrils [4]. These less crystalline regions are postulated to functionally serve to link fibrils with other wall components and thus may alter the wall structure and biomechanics. Biomechanical properties of various plant tissues under various conditions have been examined using primary shearing force, punch and dye testing or tensile strength [19]–[20]. The aims of this study were to explore the technical limitations and responses of the relative crystallinity measure to ecosystem level processes using x-ray diffraction [21]–[29]. RCI analysis was overlaid with plant species diversity, response to abiotic community boundaries and endemic plasticity. These combined data showed marked variability of relative sample crystallinity in plants across these ecological niches, but detailed quantitative determination of cellulose crystallite size, orientation and density of samples remained elusive.

Care must be taken when correlating the RCI index against plant samples. A series of detailed studies on the properties of wood cellulose have shown that RCI for pure cellulose samples is directly related to the cellulose crystallinity, but in plant samples that contain a matrix of amorphous polymers in addition to cellulose, the RCI represents the volume fraction of crystalline cellulose within the given sample [23]–[25]. The central problem in interpreting the diffractograms by the RCI value is the preferred orientation of the crystallites (texture) contributing to change in RCI [24], [27]. Therefore, RCI values as measurement of the absolute crystallinity *per se* are comparable only if the samples have the same texture, which can be altered by orientation and size of crystallites [23]–[25], [30]. It is postulated that even within the cell wall of a single cell, considerable variability in the cellulose length, orientation and absolute crystallinity may exist. Despite the problems with using x-ray diffraction to analyze plant materials [27], determination of RCI has the capacity to distinguish changes in the volume fraction of cellulose as the crystallinity of a sample. Further, established covariance of any trait against the complexity of a biological system, in particular the relatively uncharted plant biomechanical system poses many challenges. Therefore, RCI was examined in three definable experimental categories; 1) comparison of leaves, stems and roots (assembly) 2) acclimation to abiotic stress in hypocotyls, stems and leaves and 3) adaptation of RCI in leaves of a diverse group of land plants. Are results for different samples comparable? Analysis of 12 different Arabidopsis sample replicates showed very high similarity of RCI between replicates (approx 2%, Table 1). Moreover, upon examination of grasses, several wild grown Switchgrass species were analyzed from different sites and growing seasons and were not significantly different from the previous analysis. However, when defining RCI, we cannot guarantee that samples are comparable other than the determined RCI parameter is related to proportional of crystalline cellulose in the foliar sample.

Within the body plan of a single plant (Arabidopsis) there was significant variability in RCI values between the roots or stems and rosettes (Figure 3A and 3B), but not between roots and stems, suggestive of the different biomechanical architecture underlying ontogeny. For example, the RCI value was 23.4% lower in rosettes than stems. Structural requirements and allocation of cellular energy into maximal photosynthetic surface area [31] in rosettes is greatly different from the requirements of rigidity and solute transport in the stem tissue [34]. Keeping in mind that the RCI measure does not determine whether preferred orientation, size or absolute crystallinity were contributing to the lower RCI value of leaves, it is evident that RCI represents a much lower crystallinity of the leaf sample. The volume fraction of cellulose in these tissues followed a similar trend with 36% lower cellulose content in leaves than stems.

When Arabidopsis plants were grown under conditions of constant wind (thigmomorphogensis) stems had lower RCI under high wind conditions than those grown without high wind stress (Table 1). It is established that wind treatment results in decreased Young's modulus (stiffness of a material)[19], which infers a positive correlation between RCI and Young's modulus. Reports of the effect of wind on biomechanics suggest that plants are dwarfed due to investing a greater proportion of energy into mechanical reinforcement rather than surface area for photosynthesis [19], [20]. In stems, structural changes have been reported as producing “flexure fibers” or postulated high microfibrillar angle cellulose observed by Telewski [35] under conditions of high wind to provide greater flexibility. But, crystallinity for compression wood has been shown to be lower than that of normal wood simply because the share of amorphous cell wall components is larger in compression wood and the difference in crystallinity between normal wood and compression wood is modest according to Newman [36].

Experimental analysis of hypocotyls from dark grown pea plants showed that the RCI decreased compared with light grown hypocotyls (Figure 3C). The biophysical nature of etiolated tissues was very different from light grown with more than double the average length of light grown hypocotyls (3.7 cm to 9.5 cm, P<0.001) and the cellulose synthase array oriented from transverse to longitudinal when dark grown seedlings were exposed to light (within 30 min, [14] Figure 3D). Given the results of Yoshida et al. [30] and Stubicar et al. [37], the lower RCI measured in dark grown hypocotyls may be achieved by reducing the physical length dimensions of cellulose fibrils. Or, given the experiments of Andersson et al. [27] several factors associated with sample texture, including orientation, are likely to contribute. In attempting to use the leaf and non-woody biomass samples for the analysis of texture by transmission geometries, several technical challenges arose. Mounting the sample into a pressed KBr opaque disk with the sample embedded was achieved but maintaining any control over fiber orientation was not. Further, x-ray penetration through the sample was not sufficient for symmetrical transmission geometries (110 and 200A peaks were below measureable intensity). Alternatively, FTIR analysis of the light versus dark grown pea hypocotyls distinguished between the samples based on variance in the polysaccharide fingerprint region [33]. Several spectral peaks in the variance plot were assigned to cellulose (Figure 3E). We attempted to investigate this hypothesis using live cell imaging to compare the orientation, velocity, track length, rosette number and density of YFP::CESA6 particles at cortex in dark (transverse) versus after light exposure (longitudinal) arrays. However, only array orientation was found to significantly differ among treatments [14]. It was evident that longer maximum trajectories of YFP::CESA6 particles could be traced after light exposure induced reorientation to a longitudinal array (14.4 µm in dark to 26.8 µm after light exposure), but the average track lengths were not significantly different due to the large range and an inability to track through the z-focal plane. Since each tracks was the sum of multiple particles (YFP puncta) we assessed the particle number per track and found that within the dark grown hypocotyl of Arabidopsis seedlings, regardless of dark growth or brief light exposure the YFP:CESA6 density were 0.86 and 1.05 particles per mm respectively. Given this density and their bidirectional velocity of approximately 270 nm.min^−1^ [14], [16] microfibril overlap along a track would perhaps be clearer in micrographs [1].

It was evident that monocotyledons had greater foliar RCI values than the dicotyledons tested (Figure 2). Also, a trend of increasing RCI with increasing LMA and leaf length was measured (Figure 4A and 4B), which leans towards a possible allometric relationship [38]. Studies on the tensile properties of plants have shown that the grasses have 5–10 times higher tensile strength than dicots for a given LMA, which suggests that the amount of fiber itself cannot account for the difference in tensile strength (Yusuke Onoda, Personal Communication). In addition to being central to plant cell shape and morphogenesis, cell walls and their biomechanical properties are thought to play a vital role in plant defense against both biotic and abiotic stress and hence have an integral role in functional plant ecology [20]. Yet, plant biomechanics are poorly understood compared with other functional traits. Does RCI depend linearly on the crystallinity of the biomass sample? From the RCI value and experiments performed, it is arguable that the answer is no. Preferred orientation of the fibril array may in fact cause changes in the RCI consistent with finding of Andersson et al. [27] and YFP::CESA6 experiments as can the size of cellulose crystallites [30] and ball milling experiments. It is noteworthy that the possibility of exploiting RCI as a trait may exist; in that lower RCI values of plant samples were far more readily turned into fermentable sugars [22], [30]. Therefore RCI may be part of the assessment strategy useful to breeders for selection of perennial grasses with more digestible properties for forage and biofuel production.

## Materials and Methods

### Chemicals

All chemicals and reagents used were of analytical grade or higher. Authentic samples of organic acids and their salts were obtained from Sigma Aldrich, FMC BioPolymer, Fisher Scientific, Riedel den Haan and BDH as applicable.

### Statistical analysis

Hierarchical clustering, frequency distribution analysis (histogram) and analysis of variance were performed in the freeware statistical program *R* (Auckland, NZ).

### Plant Material and Sampling

Foliar samples from non-Arabidopsis plants were from plants grown in Lexington Kentucky at the University of Kentucky Arboretum on the University of Kentucky campus and an Agricultural Experimental Station research farm. Samples from 35 different plant specimens were used in experiments were as follows: *Marchantia polymorpha, Sphaeopterus cooperi, Asparagus setaceus, Sedum morganianum, Podocarpus macrophyllus, Cycas circinalis, Araucaria araucana, Equisetum hyemale, Epiphyllum oxypetalum, Aspidistra elatior, Drosera adelae, Arabidopsis thaliana, Pisum sativum, Panicum virgatum* (Cave in Rock, Alamo, Trailgrazer), *Arundo donax, Miscanthus giganteus, Miscanthus sinescens, Miscanthus saccharifolia, Cyanodon tactylon*, *Phalaris arundinacea L., Eragrostis tef, Eragrotis curvula, Tripsacum dactyloides* (PMK24, Meade Co and Jackson), *Festuca arundinacea, Spentina pectinaria, Chasmanthium latifolium, Sorghastrum nutans (Cheyenne and Rumsey), Muhlenbergia shreberi, Andropogon gerardii* (KYAG9601 and KAW) and *Sorghum bicolor.* Sampling occurred during 2007–2008 and leaf samples for the study of RCI were batch samples of 15 leaves. *Arabidopsis thaliana* cv. Columbia plants were analyzed as whole plants samples or divided into roots, shoots and leaves. These plants were grown at 22°C under a 16 h light 8 h dark regime in 3 batches of 50 plants and harvested as plant biomass. The transgenic plants expressing YFP::CESA6 were slightly different than those previously published [15]–[17] in that we extensively re-screened the T1 generation of the transformation described by Parerdez et al. [14] to isolate a homozygous allele that complemented the procuste (CESA6) mutant since the previous allele was heterozygous. We selected the homozygous allele that had functional and bright expression of the YFP.

### Characterization of Natural Variation in Relative Crystallinity by X-Ray Scattering

Samples were prepared by oven drying biomass at 60°C for 36 hours. Alternative temperatures for the drying regime were used, such as 37°C for 7 days or 80°C for 12 hours followed by 110°C for two hours, neither of which altered the RCI value measured in Arabidopsis tissue. Tissue was then homogenized using as Arthur H Thomas Co Scientific grinder (Phila, PA) equipped with a 1 mm sieve. For experiments testing finely ground biomass, homogenization was achieved by ball milling into fine powder. Biomass samples were then contained in a custom built sample holder of pressed boric acid. In brief, plant material was placed into a mold, containing a sleeve and hand pressed with a solid metal plug forming a disk shape. The sleeve and plug were removed and a boric acid (Fischer, Madison, WI) base was then formed by pouring the boric acid over the bottom and sides of the sample and applying 40,000 psi of pressure to the 40×40 mm mold using a Carver Autopellet Press (Wabash, IN). Samples were pressed to create an even horizontal surface. A Bruker-AXS Discover D8 Diffractometer (Bruker-AXS USA, Madison, WI) was used for wide angle X-ray diffraction with Cu Ka radiation generated at 30 mA and 40 kV. The experiments were carried out using Bragg-Brentano geometries (symmetrical reflection). Diffractograms were collected between 2° and 70° or 2° and 40° (for samples with little baseline drift), with 0.02° resolution and 2 s exposure time interval for each step (run time, 2 h). Sample rotation to redirect the x-ray beam diffraction site was achieved per replicate. Arabidopsis material was grown under both greenhouse and growth chamber for analysis. The data analysis was carried out using the calculation for relative crystallinity index [22] of: RCI = *I_002_−I_am_*/*I_002_*×100, where *I_002_* is the maximum peak height above baseline at approximately 22.5° and *I_am_* is the minimum peak height above the baseline at ∼18°. For assessment of experimental accuracy, the pressed samples were examined using reflective geometries at 22.5° 2-theta with the sample scanned rotationally (360°) and in an arc (90°) to obtain an intensity/spatial orientation plot of a sample for which the RCI had already been established. The range of reflective intensities was then used to estimate the accuracy of the RCI determination using a 95% cutoff across the plot range. Diffractograms were collected in Diffrac-Plus-XRD Commander software (Bruker-AXS, Karlsruhe, Germany) and minimally processed (baseline identification, noise correction, 3D display and cropping of RCI signature region) using the EVA and TexEval (Bruker-AXS Karlsruhe, Germany) software.

### Etiolation and high wind experiments

*Pisum sativum* (pea) was chosen for etiolation experiments. Changes in the cellulose relative crystallinity index were tested in hypocotyls of pea plants grown under light and dark conditions. Approximately 100 pea seedlings were germinated in soil and identical flats were halved and either grown in darkness to encourage etiolated hypocotyls or in light (16:8 light: dark) conditions for 7 days. Whole plants were then harvested, cleaned with Millipore water and leaf and root tissue removed and discarded. Hypocotyls were then pooled and oven dried to complete dryness at 80°C for 36 hours and pressed and packed in custom-built boric acid base as described above for measurement of relative crystallinity by x-ray diffraction. For high wind experiments, *Arabidopsis thaliana* Columbia plants were germinated and grown for 7 days under no wind conditions and then either exposed to wind produced by a fan at a constant average air velocity of 4.6 meters per second for a period of 14 days. All plants were container grown under growth room conditions of 16:8 light: dark for both treatments and watered regularly to avoid dehydration. Stem tissue and leaf tissues were separated and independently dried at 80°C for 36 hours prior to processing and XRD analysis as described above. Static and video imaging was performed using a Kodak M863 camera (8.2 MegaPixel) (Eastman Kodak Company, Rochester, NY).

### Leaf Mass/Area and Leaf Length Measurements Correlated With RCI

Leaf mass per unit area (LMA) measures the dry mass investment per unit of light intercepting the leaf area deployed and leaf length of all leaves was measured from the beginning of the leaf blade to the axial tip of the leaf (cm). All measurements were plotted against the RCI values in Excel (Microsoft) software.

### Confocal Microscopy

Seeds were germinated on 0.5 X MS agar for 2–4 d in darkness at 21°C by wrapping in aluminum foil. Seedlings were mounted in water between a slide and a cover slip. Once mounted, specimens were imaged in darkness, then exposed to light for 5 min and then imaged again 30 minutes later. Imaging was performed on an Olympus FV1000 laser scanning confocal microscope using a 63× N.A 1.4 oil-immersion objective. The microscope is equipped with lasers for excitation wavelengths ranging from 405–633 nm and EYFP was excited using the EYFP setting in the Olympus Fluoview software (Olympus). All image processing was performed by using Olympus Fluoview software (Olympus) and ImageJ (W. Rasband, National Institutes of Health, Bethesda, MD) software.

### FT-IR Spectroscopy

For Fourier Transform Infrared analysis (FT-IR), pre-cleaned by solvent extractions and dried pea material were pooled and homogenized by excessive milling. The powder was mixed with KBr, and pressed into 7-mm pellets. Four FT-IR spectra for each line were collected on a Thermo Nicolet Nexus 470 spectrometer (ThermoElectric Corporation, Chicago, IL) over the range 4,000–800 cm^−1^ [33]. For each spectrum, 200 scans were co added at a resolution of 8 cm^−1^ for Fourier transform processing and absorbance spectrum calculation by using OMNIC software (Thermo Nicolet, Madison, WI). Spectra were corrected for background by automatic subtraction and saved in JCAMP.DX format for further analysis. Using win-das software (Wiley, New York), spectra were baseline-corrected and were normalized and analyzed by using the analysis covariance matrix method. Figures were processed using Adobe Illustrator.

### Cellulose determinations

Cellulose contents were measured spectrophotometrically according to the protocol of [39] on homogeneous samples of ground biomass. Spectrophotometry was performed on a ThermoFischer Biomate3 (Madison, WI).
